# Profound Iron Deficiency Anemia and Irreversible Dilated Cardiomyopathy in a Child

**DOI:** 10.1155/2019/7513782

**Published:** 2019-10-10

**Authors:** Samuel G. Wittekind, Hugo R. Martinez, Chet Villa, Matthew Bacon, Adrienne M. Hammill, Angela Lorts

**Affiliations:** ^1^Cincinnati Children's Hospital Medical Center, Heart Institute, Cincinnati, OH, USA; ^2^Department of Pediatrics, University of Cincinnati College of Medicine, Cincinnati, OH, USA; ^3^Cincinnati Children's Hospital Medical Center, Division of Hematology, Cincinnati, OH, USA

## Abstract

Iron deficiency anemia has been associated with a secondary and potentially reversible cardiomyopathy. The pathophysiologic paradigm has been that the hematologic disease begets cardiac dysfunction. There may be, however, a point at which myocardial injury is irreversible in susceptible individuals. We present the case of a 4-year-old, developmentally normal, child who presented with iron deficiency anemia and a dilated cardiomyopathy with congestive heart failure. Despite appropriate correction of the anemia, the patient developed decompensated heart failure requiring milrinone therapy and eventual heart transplantation. This report will alert clinicians to the potential for irreversible adverse cardiac remodeling and the importance of close pediatric cardiology consultation and serial assessment in order to implement appropriate heart failure therapy.

## 1. Introduction

Anemia is highly prevalent in heart failure (HF) and is associated with increased morbidity in adults and children [1–3]. Iron deficiency anemia, in particular, has been implicated in the development of a secondary cardiomyopathy [4, 5] though a definitive pathogenesis has not been identified. Mechanisms linking anemia and HF might include (1) hemodilution in the setting of high-output HF, (2) elevated sympathetic tone, (3) impaired oxygen delivery and myocardial oxygen extraction, (4) renal dysfunction, and (5) poor dietary intake of micronutrients essential for myocardial function. Previous reports of high-output HF reversing with correction of the iron deficiency anemia [4, 5] are encouraging; however, such recovery should not be assumed. We present the case of a young child with severe anemia and cardiac dysfunction to highlight the diagnostic challenge of determining causation versus association. We also highlight the vital importance of differentiating high- and low-output HF states in this setting since they can change over time with major treatment implications.

## 2. Case Presentation

A previously healthy 4-year-old, 14 kg boy presented to the emergency department with several days of malaise, pallor, and tachypnea. He had a brief febrile illness one week prior to the onset of these symptoms. He was found to have severe microcytic anemia (hemoglobin 1.9 g/dL, MCV 46.4 fL) with iron deficiency (reticulocyte 4.4%, ferritin 1.1 ng/mL, iron 8 mg/dL, and RDW 35.3%) and normal micronutrient levels otherwise including selenium. Due to a poor appetite for solid food that began one year earlier, he was favoring liquids and was drinking roughly 60 fluid ounces per day of cow's milk. There was no evidence of gastrointestinal blood loss. On physical exam, he was developmentally appropriate, well perfused, and had signs of congestive HF including pitting edema of the lower extremities. The initial transthoracic echocardiogram revealed a dilated left ventricle (LV) (LVEDD 4.61 cm, *z*‐score + 5.85) with moderate LV systolic dysfunction (LVEF 33%). In addition, the left atrium (LA) was mildly dilated with an indexed volume of 39.5 mL/m^2^ (normal < 30 mL/m^2^), and there was mild mitral regurgitation (MR). A 12-lead ECG demonstrated sinus rhythm with left ventricular hypertrophy; there was no arrhythmia on telemetry. He was transfused with packed red blood cells over two days to a hemoglobin of 8.5 g/dL along with furosemide to prevent transfusion-related circulatory overload and given a replacement dose of intravenous iron dextran followed by supplemental oral ferrous sulfate. His appetite improved dramatically with correction of the anemia. Enalapril was initiated for LV reverse remodeling which he tolerated well. Screening for the common viral etiologies of myocarditis by serum PCR testing was negative. Given that he had not seen a primary care provider since he was 18 months of age, the patient was discharged home with wraparound services and close follow-up with hematology and cardiology. The discharge diagnosis was cow's milk-induced iron deficiency anemia with associated cardiac dysfunction.

**Figure 1:**
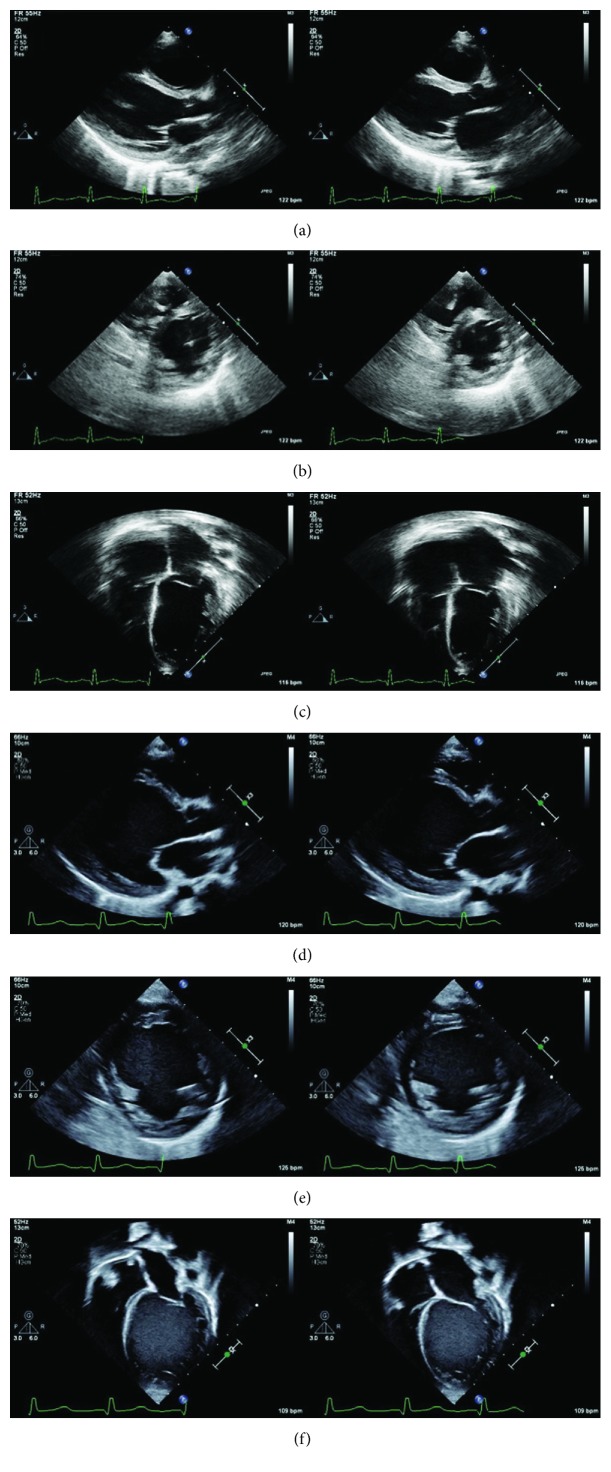
Transthoracic echocardiography (TTE) demonstrating LV size and systolic function at initial presentation (a–c) and 4 months later on milrinone therapy awaiting heart transplantation (d–f). The heart became progressively more dilated and dysfunctional over time. (a) Parasternal long axis view (PLAX) at end-diastole (left) and end-systole (right). (b) Parasternal short axis (PSAX) view at end-diastole (left) and end-systole (right). (c) Apical 4-chamber (A4C) view at end-diastole (left) and end-systole (right). (d) PLAX view at end-diastole (left) and end-systole (right). (e) PSAX view at end-diastole (left) and end-systole (right). (f) A4C view at end-diastole (left) and end-systole (right).

The patient returned to the cardiology clinic 2 weeks later with signs of worsening congestive HF including fatigue, abdominal discomfort with decreased appetite, weight up 2 kg, liver edge 4 cm below the right costal margin, a serum B-type natriuretic peptide of 9,724 pg/mL, and a low mixed venous oxygen saturation. The echocardiogram demonstrated signs of worsening dilated cardiomyopathy (DCM) including an indexed LA volume 78.9 ml/m^2^, LVEDD 4.96 cm (*z*‐score + 6.66 H), LVEF 15%, and moderate MR. The hemoglobin had normalized to 12.3 g/dL by this time. He was admitted for intravenous diuretics and milrinone infusion for decompensated, low-output HF. Over a period of 2 months, he remained symptomatic, the LV further dilated without an improvement in systolic function, and he developed signs of congestive hepatopathy. Serial echocardiographic images demonstrating the progressive LV dilation are shown in Figure 1. Milrinone therapy was continued while he was listed status 1B for heart transplantation. He received an orthotopic heart transplant 2 months after listing (4.5 months after initial presentation) with an excellent outcome.

Histopathology on the explanted heart showed essentially normal appearing myocardial fibers with patchy, subendocardial areas of mild hypertrophy. There was no suggestion of myocardial fiber disarray, interstitial fibrosis, storage disorder, or inflammation. Electron microscopy demonstrated normal myocardial ultrastructure with no evidence of mitochondriopathy. Genetic testing for 47 genes implicated in DCM (Invitae, San Francisco, CA) was negative for known pathologic variants. Echocardiographic screenings of first-degree relatives have been negative to date.

## 3. Discussion

There are unique features of this case that challenge the pathophysiologic paradigm of iron deficiency itself leading to cardiac dysfunction. Firstly, his age at presentation makes him an outlier for the cow's milk-induced iron deficiency anemia commonly seen in toddlers between the ages of 1 and 3 years [6]. The chronic refusal of solid foods may have been a symptom of HF as it is well known that feeding difficulties and associated abdominal discomfort are often how HF presents in children [7]. Next, the LV was considerably dilated at presentation beyond what is typical for iron deficiency cardiomyopathy. Additionally, the LA was dilated at the time which is evidence of chronically elevated LV filling pressure [8]. Finally, there was rapid disease progression to the point of requiring advanced HF therapies after appropriate correction of the anemia. Notably, the lack of impressive histopathologic findings as well as the negative gene panel is common in pediatric DCM [9] and should not be taken as strong evidence against a primary cardiomyopathy. It seems most likely from the available evidence that iron deficiency anemia and DCM progressed in tandem and compounded each other in this case.

This case highlights the importance of regular pediatric primary care visits to review diet and screen for anemia in high-risk patients [6]. To our knowledge, this is the first report of a patient requiring advanced HF therapy despite appropriate correction of profound iron deficiency anemia. An expectation that the heart will recover may not be valid in all cases; there may be a “point of no return” in this type of cardiomyopathy. Furthermore, suspicion for a primary cardiomyopathy should be heightened when the heart is significantly dilated and dysfunctional at presentation since there are major prognostic and treatment implications. After correction of the anemia in this clinical scenario, close pediatric cardiology consultation and serial assessment of cardiac function can identify decompensation and promptly institute life-saving HF therapy when needed.

## Abbreviations

DCM: Dilated cardiomyopathy
HF: Heart failure
kg: Kilogram
LA: Left atrium
LV: Left ventricle
LVEDD: Left ventricular end-diastolic diameter
LVEF: Left ventricular ejection fraction
MCV: Mean corpuscular volume
MR: Mitral regurgitation
RDW: Red cell distribution width.
